# Crystal structure of (*E*)-2-(4-chloro­benzyl­idene)-3,4-di­hydro­naphthalen-1(2*H*)-one: a second monoclinic polymorph

**DOI:** 10.1107/S2056989015016151

**Published:** 2015-09-12

**Authors:** Muhammad Haroon, Tashfeen Akhtar, Muhammad Nawaz Tahir

**Affiliations:** a Department of Chemistry, Mirpur University of Science and Technology (MUST), Mirpur, Azad Jammu and Kashmir, Pakistan; b Department of Physics, University of Sargodha, Sargodha, Punjab, Pakistan

**Keywords:** crystal structure, α-tetra­lone, C—H⋯π inter­actions

## Abstract

The title compound, C_17_H_13_ClO, is the second monoclinic polymorph to crystallize in the space group *P*2_1_/*c*. The first polymorph crystallized with two independent mol­ecules in the asymmetric unit [Bolognesi *et al.* (1975 ▸). *Acta Cryst.* A**31**, S119; *Z*′ = 2; no atomic coordinates available], whereas the title compound has *Z*′ = 1. In the title polymorph, the dihedral angle between the plane of the benzene ring of the tetra­lone moiety and that of the 4-chloro­benzyl ring is 52.21 (11)°. The cyclo­hex-2-en-1-one ring of the tetra­lone moiety has a screw-boat conformation. In the crystal, mol­ecules are liked by pairs of C—H⋯π inter­actions forming inversion dimers. There are no other significant inter­molecular inter­actions present.

## Related literature   

For a brief description of the first monoclinic polymorph of the title compound, see: Bolognesi *et al.* (1975 ▸). For the crystal structures of related compounds, see: Asiri *et al.* (2012 ▸); Dimmock *et al.* (2002 ▸); Oloo *et al.* (2002 ▸). For the synthesis, see: Kerbal *et al.* (1988 ▸).
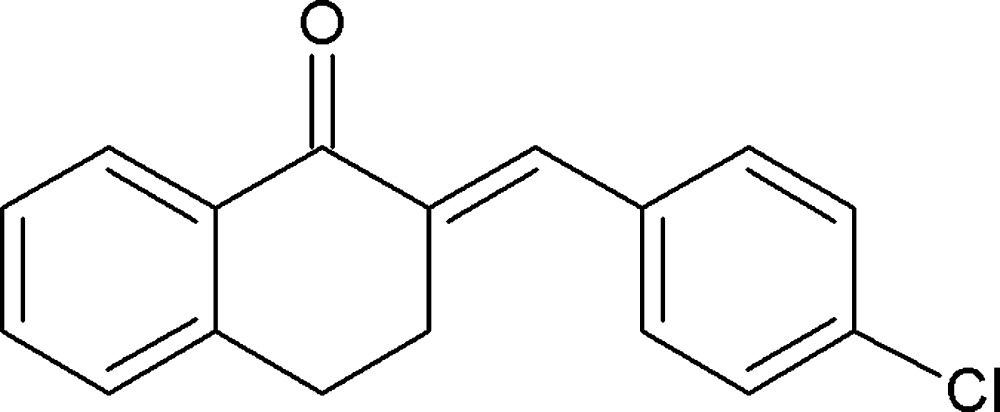


## Experimental   

### Crystal data  

C_17_H_13_ClO
*M* *_r_* = 268.72
Monoclinic, 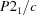

*a* = 13.3791 (8) Å
*b* = 14.9352 (10) Å
*c* = 6.7849 (3) Å
β = 93.968 (3)°
*V* = 1352.51 (14) Å^3^
*Z* = 4
Mo *K*α radiation
μ = 0.27 mm^−1^
*T* = 296 K
0.38 × 0.30 × 0.26 mm

### Data collection  

Bruker Kappa APEXII CCD diffractometer
Absorption correction: multi-scan (*SADABS*; Bruker, 2007 ▸) *T*_min_ = 0.906, *T*_max_ = 0.930
11640 measured reflections
2954 independent reflections
1901 reflections with *I* > 2σ(*I*)
*R*_int_ = 0.029

### Refinement  

*R*[*F* ^2^ > 2σ(*F* ^2^)] = 0.051
*wR*(*F* ^2^) = 0.170
*S* = 1.05
2954 reflections
172 parameters
H-atom parameters constrained
Δρ_max_ = 0.37 e Å^−3^
Δρ_min_ = −0.19 e Å^−3^

### 

Data collection: *APEX2* (Bruker, 2007 ▸); cell refinement: *SAINT* (Bruker, 2007 ▸); data reduction: *SAINT*; program(s) used to solve structure: *SHELXS97* (Sheldrick, 2008 ▸); program(s) used to refine structure: *SHELXL2014* (Sheldrick, 2015 ▸); molecular graphics: *ORTEP-3 for Windows* (Farrugia, 2012 ▸) and *PLATON* (Spek, 2009 ▸); software used to prepare material for publication: *WinGX* (Farrugia, 2012 ▸) and *PLATON*.

## Supplementary Material

Crystal structure: contains datablock(s) global, I. DOI: 10.1107/S2056989015016151/su5197sup1.cif

Structure factors: contains datablock(s) I. DOI: 10.1107/S2056989015016151/su5197Isup2.hkl

Supporting information file. DOI: 10.1107/S2056989015016151/su5197Isup3.cml

**Fig. 1.**
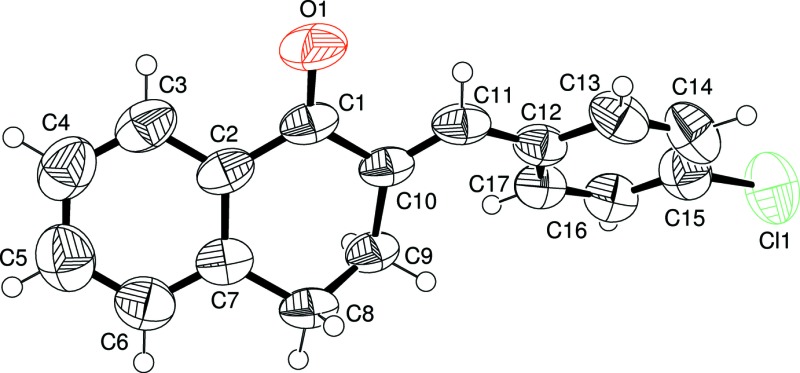
View of the molecular structure of the title compound, with atom labelling. Displacement ellipsoids are drawn at the 50% probability level.

. DOI: 10.1107/S2056989015016151/su5197fig1.tif
View of the mol­ecular structure of the title compound, with atom labelling. Displacement ellipsoids are drawn at the 50% probability level.

CCDC reference: 1421217

Additional supporting information:  crystallographic information; 3D view; checkCIF report

## Figures and Tables

**Table 1:** Hydrogen-bond geometry (, ) *Cg*1 is the centroid of the C2C7 ring.

*D*H*A*	*D*H	H*A*	*D* *A*	*D*H*A*
C13H13*Cg*1^i^	0.93	2.86	3.552(2)	132

Symmetry code: (i) 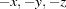.

## supplementary crystallographic information

### S1. Comments

The crystal structure of 2-(*p*-chloro­benzyl­idene)-tetral-1-one (Bolognesi *et al.*, 1975) is the first monoclinic polymorph of the title compound, however no atomic coordinates were reported. The crystal structures of the related structures 2-(2,4-di­chloro­phenyl­methyl­ene)-1- tetra­lone (Oloo *et al.*, 2002), 2-[(*E*)-4-meth­oxy­benzyl­idene]-1,2,3,4-tetra­hydro­naphthalen-1-one (Asiri *et al.*, 2012), and 2-(3,4-di­chloro­phenyl­methyl­ene)-1-tetra­lone (Dimmock *et al.*, 2002) have been published.

The molecular structure of the title polymorph is illustrated in Fig. 1. The benzene ring (C2–C7) of tetra­lone (systematic name: 3,4-di­hydro­naphthalen-1(2*H*)-one) and the mean plane of part of the 4-chloro­benzyl­idene (C11–C17/Cl1) moiety are inclined to one another by 52.03 (6)°. The cyclo­hex-2-en-1-one ring, (C1/C2/C7–C10), has puckering amplitude (Q) = 0.471 (2) Å, and θ = 65.6 (2)° and φ = 210.5 (3)°, and can be described as having a screw-boat conformation.

In the crystal, molecules are liked by pairs of C—H···π inter­actions forming inversion dimers (Table 1). There are no other significant inter­molecular inter­actions present.

### S2. Synthesis and crystallization

The synthesis of the title compound was carried out following a published procedure (Kerbal *et al.*, 1988), *viz.* by a condensation of equimolar amounts of 4-chloro­benzaldehyde and α-tetra­lone using sodium hydroxide in methanol (yield; 87%; m.p.: 426–428 K). The synthesized compound was crystallized in tetra­hydro­furan under slow evaporation yielding light-orange prismatic crystals.

### S3. Refinement

Crystal data, data collection and structure refinement details are summarized in Table 2. The H-atoms were positioned geometrically (C–H = 0.93–0.97 Å) and refined as riding with *U*_iso_(H) = 1.2*U*_eq_(C).

### Crystal data

**Table:**

C_17_H_13_ClO	*F*(000) = 560
*M**_r_* = 268.72	*D*_x_ = 1.320 Mg m^−^^3^
Monoclinic, *P*2_1_/*c*	Mo *K*α radiation, λ = 0.71073 Å
*a* = 13.3791 (8) Å	Cell parameters from 1901 reflections
*b* = 14.9352 (10) Å	θ = 2.7–27.0°
*c* = 6.7849 (3) Å	µ = 0.27 mm^−^^1^
β = 93.968 (3)°	*T* = 296 K
*V* = 1352.51 (14) Å^3^	Prism, light orange
*Z* = 4	0.38 × 0.30 × 0.26 mm

### Data collection

**Table:**

Bruker Kappa APEXII CCD diffractometer	2954 independent reflections
Radiation source: fine-focus sealed tube	1901 reflections with *I* > 2σ(*I*)
Graphite monochromator	*R*_int_ = 0.029
Detector resolution: 7.70 pixels mm^-1^	θ_max_ = 27.0°, θ_min_ = 2.7°
ω scans	*h* = −17→17
Absorption correction: multi-scan (*SADABS*; Bruker, 2007)	*k* = −19→18
*T*_min_ = 0.906, *T*_max_ = 0.930	*l* = −8→8
11640 measured reflections	

### Refinement

**Table:**

Refinement on *F*^2^	Primary atom site location: structure-invariant direct methods
Least-squares matrix: full	Secondary atom site location: difference Fourier map
*R*[*F*^2^ > 2σ(*F*^2^)] = 0.051	Hydrogen site location: inferred from neighbouring sites
*wR*(*F*^2^) = 0.170	H-atom parameters constrained
*S* = 1.05	*w* = 1/[σ^2^(*F*_o_^2^) + (0.0931*P*)^2^ + 0.1261*P*] where *P* = (*F*_o_^2^ + 2*F*_c_^2^)/3
2954 reflections	(Δ/σ)_max_ < 0.001
172 parameters	Δρ_max_ = 0.37 e Å^−^^3^
0 restraints	Δρ_min_ = −0.18 e Å^−^^3^

### Special details

**Table:**

Geometry. All e.s.d.'s (except the e.s.d. in the dihedral angle between two l.s. planes) are estimated using the full covariance matrix. The cell e.s.d.'s are taken into account individually in the estimation of e.s.d.'s in distances, angles and torsion angles; correlations between e.s.d.'s in cell parameters are only used when they are defined by crystal symmetry. An approximate (isotropic) treatment of cell e.s.d.'s is used for estimating e.s.d.'s involving l.s. planes.
Refinement. Refinement of *F*^2^ against ALL reflections. The weighted *R*-factor *wR* and goodness of fit *S* are based on *F*^2^, conventional *R*-factors *R* are based on *F*, with *F* set to zero for negative *F*^2^. The threshold expression of *F*^2^ > σ(*F*^2^) is used only for calculating *R*-factors(gt) *etc*. and is not relevant to the choice of reflections for refinement. *R*-factors based on *F*^2^ are statistically about twice as large as those based on *F*, and *R*-factors based on ALL data will be even larger.

### Fractional atomic coordinates and isotropic or equivalent isotropic displacement parameters (Å^2^)

**Table:**

	*x*	*y*	*z*	*U*_iso_*/*U*_eq_	
Cl1	−0.45111 (6)	0.12595 (6)	0.64763 (13)	0.1115 (4)	
O1	0.01032 (15)	0.14379 (12)	−0.1910 (2)	0.0905 (6)	
C1	0.04921 (18)	0.13078 (12)	−0.0242 (3)	0.0592 (5)	
C2	0.15886 (18)	0.13063 (12)	0.0132 (3)	0.0594 (5)	
C3	0.2190 (2)	0.16509 (15)	−0.1286 (4)	0.0758 (7)	
H3	0.1893	0.1867	−0.2471	0.091*	
C4	0.3206 (2)	0.16757 (17)	−0.0960 (5)	0.0903 (8)	
H4	0.3597	0.1910	−0.1918	0.108*	
C5	0.3657 (2)	0.13533 (18)	0.0791 (5)	0.0947 (9)	
H5	0.4350	0.1373	0.1021	0.114*	
C6	0.3064 (2)	0.09969 (16)	0.2216 (4)	0.0767 (7)	
H6	0.3369	0.0774	0.3389	0.092*	
C7	0.20322 (18)	0.09703 (13)	0.1913 (3)	0.0597 (5)	
C8	0.13788 (16)	0.05948 (14)	0.3412 (3)	0.0638 (6)	
H8A	0.1739	0.0605	0.4703	0.077*	
H8B	0.1220	−0.0024	0.3086	0.077*	
C9	0.04115 (17)	0.11253 (13)	0.3489 (3)	0.0588 (5)	
H9A	−0.0018	0.0835	0.4390	0.071*	
H9B	0.0564	0.1721	0.3992	0.071*	
C10	−0.01328 (17)	0.11966 (12)	0.1487 (3)	0.0550 (5)	
C11	−0.11281 (18)	0.11818 (12)	0.1126 (3)	0.0599 (6)	
H11	−0.1350	0.1175	−0.0203	0.072*	
C12	−0.19169 (17)	0.11744 (12)	0.2505 (3)	0.0572 (5)	
C13	−0.28306 (18)	0.07683 (15)	0.1952 (3)	0.0736 (6)	
H13	−0.2913	0.0487	0.0729	0.088*	
C14	−0.36166 (18)	0.07709 (18)	0.3162 (4)	0.0859 (8)	
H14	−0.4212	0.0477	0.2782	0.103*	
C15	−0.3506 (2)	0.12143 (16)	0.4938 (4)	0.0779 (7)	
C16	−0.26219 (17)	0.16368 (14)	0.5526 (3)	0.0689 (6)	
H16	−0.2559	0.1942	0.6723	0.083*	
C17	−0.18321 (17)	0.16064 (13)	0.4338 (3)	0.0614 (5)	
H17	−0.1229	0.1878	0.4760	0.074*	

### Atomic displacement parameters (Å^2^)

**Table:**

	*U*^11^	*U*^22^	*U*^33^	*U*^12^	*U*^13^	*U*^23^
Cl1	0.0757 (5)	0.1284 (7)	0.1334 (8)	0.0051 (4)	0.0296 (5)	−0.0236 (5)
O1	0.1112 (14)	0.1190 (15)	0.0393 (8)	−0.0036 (11)	−0.0092 (8)	0.0101 (8)
C1	0.0875 (16)	0.0509 (11)	0.0383 (10)	−0.0005 (10)	−0.0030 (9)	0.0017 (8)
C2	0.0872 (15)	0.0442 (10)	0.0469 (10)	0.0026 (9)	0.0066 (10)	0.0004 (8)
C3	0.103 (2)	0.0587 (13)	0.0674 (14)	0.0112 (12)	0.0194 (13)	0.0091 (10)
C4	0.103 (2)	0.0666 (15)	0.105 (2)	0.0074 (14)	0.0359 (16)	0.0120 (14)
C5	0.0834 (19)	0.0756 (17)	0.126 (3)	0.0051 (14)	0.0139 (18)	−0.0056 (16)
C6	0.0839 (18)	0.0650 (14)	0.0801 (16)	0.0072 (12)	−0.0013 (13)	−0.0022 (12)
C7	0.0798 (15)	0.0456 (10)	0.0529 (11)	0.0036 (9)	−0.0008 (10)	−0.0052 (8)
C8	0.0849 (15)	0.0601 (12)	0.0446 (10)	0.0039 (10)	−0.0093 (9)	0.0068 (9)
C9	0.0785 (14)	0.0581 (11)	0.0387 (9)	−0.0024 (10)	−0.0037 (9)	0.0037 (8)
C10	0.0790 (15)	0.0441 (10)	0.0404 (10)	−0.0013 (9)	−0.0070 (9)	0.0016 (7)
C11	0.0843 (16)	0.0474 (11)	0.0453 (10)	0.0016 (9)	−0.0137 (10)	−0.0023 (8)
C12	0.0691 (13)	0.0440 (10)	0.0563 (11)	0.0052 (8)	−0.0109 (9)	−0.0017 (8)
C13	0.0747 (15)	0.0690 (14)	0.0740 (14)	0.0063 (12)	−0.0172 (12)	−0.0173 (11)
C14	0.0631 (15)	0.0827 (17)	0.109 (2)	0.0007 (12)	−0.0133 (14)	−0.0218 (15)
C15	0.0698 (16)	0.0701 (15)	0.0940 (18)	0.0099 (11)	0.0064 (13)	−0.0040 (13)
C16	0.0787 (16)	0.0575 (12)	0.0696 (13)	0.0069 (11)	−0.0009 (11)	−0.0084 (10)
C17	0.0744 (14)	0.0477 (11)	0.0607 (12)	−0.0011 (9)	−0.0068 (10)	−0.0051 (9)

### Geometric parameters (Å, º)

**Table:**

Cl1—C15	1.759 (3)	C8—H8B	0.9700
O1—C1	1.227 (2)	C9—C10	1.500 (3)
C1—C2	1.471 (3)	C9—H9A	0.9700
C1—C10	1.496 (3)	C9—H9B	0.9700
C2—C3	1.394 (3)	C10—C11	1.337 (3)
C2—C7	1.402 (3)	C11—C12	1.458 (3)
C3—C4	1.363 (4)	C11—H11	0.9300
C3—H3	0.9300	C12—C13	1.393 (3)
C4—C5	1.382 (4)	C12—C17	1.399 (3)
C4—H4	0.9300	C13—C14	1.378 (3)
C5—C6	1.398 (4)	C13—H13	0.9300
C5—H5	0.9300	C14—C15	1.374 (4)
C6—C7	1.383 (3)	C14—H14	0.9300
C6—H6	0.9300	C15—C16	1.376 (3)
C7—C8	1.495 (3)	C16—C17	1.373 (3)
C8—C9	1.521 (3)	C16—H16	0.9300
C8—H8A	0.9700	C17—H17	0.9300
			
O1—C1—C2	120.87 (19)	C8—C9—H9A	109.3
O1—C1—C10	121.1 (2)	C10—C9—H9B	109.3
C2—C1—C10	117.97 (16)	C8—C9—H9B	109.3
C3—C2—C7	119.8 (2)	H9A—C9—H9B	108.0
C3—C2—C1	119.8 (2)	C11—C10—C1	117.49 (17)
C7—C2—C1	120.46 (18)	C11—C10—C9	125.41 (19)
C4—C3—C2	121.0 (2)	C1—C10—C9	117.09 (19)
C4—C3—H3	119.5	C10—C11—C12	129.69 (18)
C2—C3—H3	119.5	C10—C11—H11	115.2
C3—C4—C5	120.1 (3)	C12—C11—H11	115.2
C3—C4—H4	120.0	C13—C12—C17	117.1 (2)
C5—C4—H4	120.0	C13—C12—C11	119.44 (18)
C4—C5—C6	119.5 (3)	C17—C12—C11	123.31 (19)
C4—C5—H5	120.2	C14—C13—C12	122.0 (2)
C6—C5—H5	120.2	C14—C13—H13	119.0
C7—C6—C5	121.1 (2)	C12—C13—H13	119.0
C7—C6—H6	119.4	C15—C14—C13	119.0 (2)
C5—C6—H6	119.4	C15—C14—H14	120.5
C6—C7—C2	118.5 (2)	C13—C14—H14	120.5
C6—C7—C8	122.29 (19)	C14—C15—C16	120.9 (2)
C2—C7—C8	119.2 (2)	C14—C15—Cl1	119.9 (2)
C7—C8—C9	111.51 (16)	C16—C15—Cl1	119.2 (2)
C7—C8—H8A	109.3	C17—C16—C15	119.8 (2)
C9—C8—H8A	109.3	C17—C16—H16	120.1
C7—C8—H8B	109.3	C15—C16—H16	120.1
C9—C8—H8B	109.3	C16—C17—C12	121.2 (2)
H8A—C8—H8B	108.0	C16—C17—H17	119.4
C10—C9—C8	111.52 (17)	C12—C17—H17	119.4
C10—C9—H9A	109.3		
			
O1—C1—C2—C3	14.6 (3)	C2—C1—C10—C11	−178.76 (17)
C10—C1—C2—C3	−162.05 (18)	O1—C1—C10—C9	−174.51 (19)
O1—C1—C2—C7	−166.10 (19)	C2—C1—C10—C9	2.2 (2)
C10—C1—C2—C7	17.2 (3)	C8—C9—C10—C11	143.7 (2)
C7—C2—C3—C4	−0.7 (3)	C8—C9—C10—C1	−37.3 (2)
C1—C2—C3—C4	178.6 (2)	C1—C10—C11—C12	−173.08 (18)
C2—C3—C4—C5	0.3 (4)	C9—C10—C11—C12	5.9 (3)
C3—C4—C5—C6	0.4 (4)	C10—C11—C12—C13	−151.1 (2)
C4—C5—C6—C7	−0.7 (4)	C10—C11—C12—C17	33.0 (3)
C5—C6—C7—C2	0.3 (3)	C17—C12—C13—C14	−1.3 (3)
C5—C6—C7—C8	−179.9 (2)	C11—C12—C13—C14	−177.4 (2)
C3—C2—C7—C6	0.4 (3)	C12—C13—C14—C15	2.3 (4)
C1—C2—C7—C6	−178.84 (19)	C13—C14—C15—C16	−1.1 (4)
C3—C2—C7—C8	−179.42 (18)	C13—C14—C15—Cl1	177.9 (2)
C1—C2—C7—C8	1.3 (3)	C14—C15—C16—C17	−0.9 (4)
C6—C7—C8—C9	142.9 (2)	Cl1—C15—C16—C17	180.00 (17)
C2—C7—C8—C9	−37.3 (3)	C15—C16—C17—C12	1.9 (3)
C7—C8—C9—C10	54.1 (2)	C13—C12—C17—C16	−0.8 (3)
O1—C1—C10—C11	4.6 (3)	C11—C12—C17—C16	175.17 (19)

### Hydrogen-bond geometry (Å, º)

Cg1 is the centroid of the C2–C7 ring.

**Table:**

*D*—H···*A*	*D*—H	H···*A*	*D*···*A*	*D*—H···*A*
C13—H13···*Cg*1^i^	0.93	2.86	3.552 (2)	132

Symmetry code: (i) −*x*, −*y*, −*z*.

## References

[bb1] Asiri, A. M., Faidallah, H. M., Zayed, M. E. M., Ng, S. W. & Tiekink, E. R. T. (2012). *Acta Cryst.* E**68**, o2190. 10.1107/S160053681202805X PMC3393990 22798855

[bb2] Bolognesi, M., Coda, A., Corsico, A. C. & Desimoni, G. (1975). *Acta Cryst.* A**31**, S119.

[bb3] Bruker (2007). *APEX2*, *SAINT* and *SADABS*. Bruker AXS Inc., Madison, Wisconsin, USA.

[bb4] Dimmock, J. R., Zello, G. A., Oloo, E. O., Quail, J. W., Kraatz, H.-B., Perjési, P., Aradi, F., Takács-Novák, K., Allen, T. M., Santos, C. L., Balzarini, J., De Clercq, E. & Stables, J. P. (2002). *J. Med. Chem.* **45**, 3103–3111. 10.1021/jm010559p 12086496

[bb5] Farrugia, L. J. (2012). *J. Appl. Cryst.* **45**, 849–854.

[bb6] Kerbal, A., Tshiamala, K., Vebrel, J. & Lauds, B. (1988). *Bull. Soc. Chim. Belg.* **97**, 149–162.

[bb7] Oloo, E. O., Quail, J. W., Perjési, P. & Dimmock, J. R. (2002). *Acta Cryst.* E**58**, o580–o581.

[bb8] Sheldrick, G. M. (2008). *Acta Cryst.* A**64**, 112–122. 10.1107/S0108767307043930 18156677

[bb9] Sheldrick, G. M. (2015). *Acta Cryst.* C**71**, 3–8.

[bb10] Spek, A. L. (2009). *Acta Cryst.* D**65**, 148–155. 10.1107/S090744490804362X PMC2631630 19171970

