# Supplementary figures and images for: Crystal structure of (E)-2-(4-chloro­benzyl­idene)-3,4-di­hydro­naphthalen-1(2H)-one: a second monoclinic polymorph
Source: Acta Crystallogr E Crystallogr Commun. 2015 Sep 12;71(Pt 10):o741–2. doi: 10.1107/S2056989015016151 (PMC4647395; doi:10.1107/S2056989015016151)

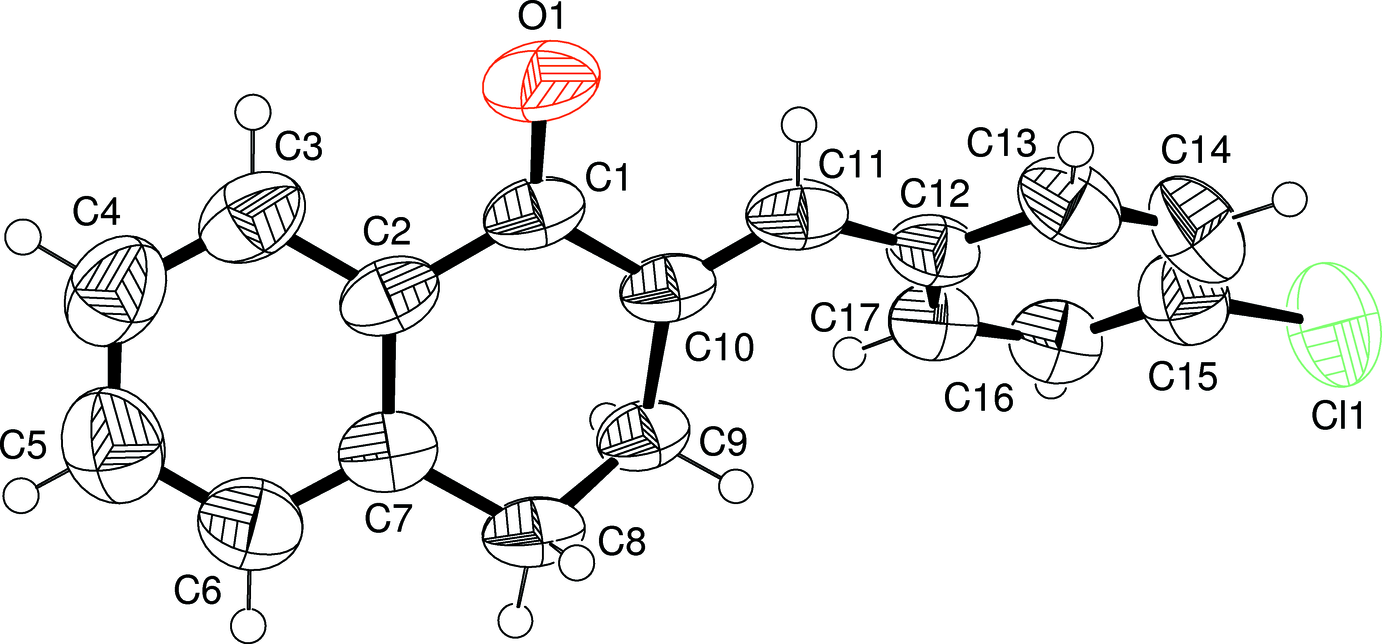

Supplement: Supplementary file 4 [file e-71-0o741-fig1.tif]
